# The Prevalence and Characteristics of Emergency Medicine Patient Use of New Media

**DOI:** 10.2196/mhealth.4438

**Published:** 2015-07-08

**Authors:** Lori Ann Post, Federico E Vaca, Brian J Biroscak, James Dziura, Cynthia Brandt, Steven L Bernstein, Richard Taylor, Liudvikas Jagminas, Gail D'Onofrio

**Affiliations:** ^1^ Yale School of Medicine Department of Emergency Medicine Yale University New Haven, CT United States; ^2^ Beth Israel Deaconess Medical Center Department of Emergency Medicine Harvard University Boston, MA United States

**Keywords:** medical informatics, new media, health care services, personal health management, mobile phones

## Abstract

**Background:**

Little is known about “new media” use, defined as media content created or consumed on demand on an electronic device, by patients in emergency department (ED) settings. The application of this technology has the potential to enhance health care beyond the index visit.

**Objective:**

The objectives are to determine the prevalence and characteristics of ED patients’ use of new media and to then define and identify the potential of new media to transcend health care barriers and improve the public’s health.

**Methods:**

Face-to-face, cross-sectional surveys in Spanish and English were given to 5,994 patients who were sequentially enrolled from July 12 to August 30, 2012. Data were collected from across a Southern Connecticut health care system’s 3 high-volume EDs for 24 hours a day, 7 days a week for 6 weeks. The EDs were part of an urban academic teaching hospital, an urban community hospital, and an academic affiliate hospital.

**Results:**

A total of 5,994 (89% response rate) ED patients reported identical ownership of cell phones (85%, *P*<.001) and smartphones (51%, *P*<.001) that were used for calling (99%, *P*<.001). The older the patient, however, the less likely it was that the patient used the phone for texting (96% vs 16%, *P*<.001). Income was positively associated with smartphone ownership (*P*<.001) and the use of health apps (*P*>.05) and personal health records (*P*<.001). Ownership of iPhones compared to Android phones were similar (44% vs 45%, *P*<.05). Race and ethnicity played a significant role in texting and smartphone ownership, with Hispanics reporting the highest rates of 79% and 56%, respectively, followed by black non-Hispanics at 77% and 54%, respectively, and white non-Hispanics at 65% and 42%, respectively (*P*<.05).

**Conclusions:**

There is a critical mass of ED patients who use new media. Older persons are less comfortable texting and using smartphone apps. Income status has a positive relationship with smartphone ownership and use of smartphone apps. Regardless of income, however, texting and ownership of smartphones was highest for Latinos and black non-Latinos. These findings have implications for expanding health care beyond the ED visit through the use of cell phones, smartphones, texting, the Internet, and health care apps to improve the health of the public.

## Introduction

New media is part of the communication science lexicon—yet it is frequently omitted from the health care literature and often is incorrectly interchanged with cell phones. “New media” is defined as media content created or consumed on demand on an electronic device (eg, mobile phones, computers, tablets, etc) [1-8]. In contrast, simple cell phone technology does not support health apps or Web browsing for health information. While most cell phones also have other communication modes beyond a simple telephone, such as texting, there is an age cohort effect whereby the elderly population is more likely to only use the phone features because that population is less comfortable texting or using mobile phone apps [9-15]. Thus, cell phones must be thought of as a subcategory of new media and distinct from mobile phones. New media has unrealized potential to improve health outcomes compared to traditional or legacy media (eg, print materials, radio, television, etc) [16-23].

According to Jenkins, new media can be thought of “as the convergence of 3 concepts—media convergence, participatory culture, and collective intelligence” [24]. With new media, consumers “interact with” a digital device as opposed to being “exposed to” legacy media, which is passive media spectatorship [24-29]. Therefore, new media has a greater potential to improve patient care and health outcomes [13,30-34]. Mobile phones, tablets, laptops, and desktops allow the consumer to search health information repositories, or “collective intelligence,” related to their health condition [35-37]. “Media convergence” refers to how patients interact with each other or experts [38-41] (eg, chat rooms for women with breast cancer) [42-45]. And a “participatory culture” allows active engagement in treatment [46-48] (eg, messaging medication adherence or provider communication) [49-51]. Engaged patients experience better health outcomes and higher satisfaction [52-58]. The purpose of this study is to improve our understanding of the prevalence, uses, and typology of new media in the emergency department (ED) care setting [59-65]. We theorize that if a critical mass of patients are using new media, it may drive a paradigm shift in health care delivery by enhancing care beyond the ED visit.

## Methods

### Overview

We designed and administered a cross-sectional survey of patients presenting to 3 EDs in southern Connecticut that are part of the Yale-New Haven Health System (YNHHS). Data were collected over 24 hours a day, 7 days a week for a total of 6 weeks. During the study period, the annual census for Yale-New Haven Hospital York Street Campus, an urban academic teaching hospital, was approximately 81,000 adult visits per year and serves a population that is 52% white, 28% black, and 18% Hispanic, with 40% receiving Medicaid. Bridgeport Hospital, an urban academic affiliate of YNHHS, receives approximately 45,000 adult visits per year and serves a population that is 44% white, 38% black, and 15% Hispanic, with 46% receiving Medicaid. The annual census for the Saint Raphael Campus ED, best described as an urban community ED, was approximately 45,000 visits per year and serves a population that is 36% white, 31% black, and 34% Hispanic, with 50% receiving Medicaid.

### Selection of Participants

**Figure 1:**
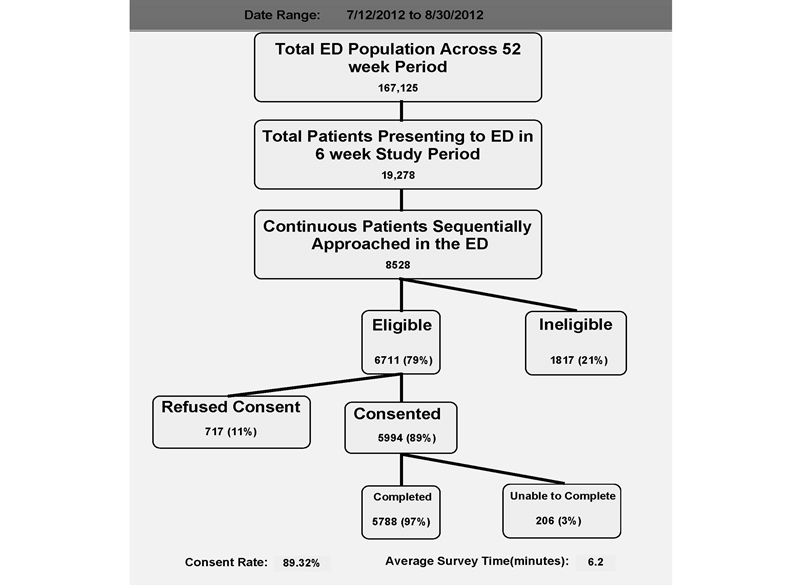
Patient flow diagram.

Research assistants (RAs) enrolled patients presenting to 1 of the 3 EDs. Twenty-two trained RAs enrolled patients on every ED shift, 24 hours a day, 7 days a week, during a 6-week period (July 12 to August 30, 2012). Patients were excluded if they were 17 years of age or younger; alcohol or drug impaired; had a condition that precluded interview; were in police custody; had active psychosis, suicidal, or homicidal ideation; or were unwilling to consent. RAs entered patient data into the electronic data capture system based on time of patient arrival (Figure 1). The institutional review board of each participating hospital approved all study procedures.

### Data Collection and Analysis

Our research consortium reviewed and selected questions from the information technology study conducted at Brown University’s ED [66], by the Department of Veterans Affairs [67], and some instruments from health communication literature [68]. Our multidisciplinary research group consisted of individuals with expertise in informatics, emergency medicine, bioinformatics, engineering, and social sciences who recommended validated questions to include on the survey instrument based on their specific areas, such as media usage [69,70], substance abuse [71,72], tobacco use [73,74], the elderly [75-81], public health records [82], veterans [83], and ethnic minorities [84-86]. The survey was derived from other validated survey or screening questionnaires and new media surveys in combination with original questions specific to the ED, health care, and patient populations. Participants were asked a series of questions representing a number of domains, such as: (1) new media technology ownership (eg, “Do you own a cell phone?”); (2) new media use (eg, “What do you use your cell phone for? Check ALL that apply.”); (3) type of technology owned (eg, “Is your cell phone a mobile phone (eg, iPhone, Blackberry, Android?”)); and (4) frequency of use (eg, “How often do you use your cell phone for text messaging?”). Contingent on answers to these prior questions, participants were asked about new media behaviors such as: (1) seeking health information (eg, “Do you use your cell phone to look up health information?”); and (2) tracking or managing one’s health (eg, “Do you use a software application on your phone to help you track or manage your health?”). The survey ended with the collection of the following demographic data: age, gender, ethnicity, race, preferred language, highest level of education completed, rural/urban status, and annual household income.

The survey was pilot tested over the course of 1 month (with observers) and tested for fourth grade Flesch-Kincaid readability. Some data regarding race were missing (<1%) due to confusion between “race” and “ethnicity.” Thus, participants who reported Latino/Hispanic as a racial category were corrected using hot deck imputation [87-98].

We compared ED patients’ new media use between 3 urban EDs in southern Connecticut. The survey was conducted in English and Spanish. We derived point estimates with 95% confidence intervals (CI) using the normal-theory method for a binomial parameter. Variables of interest include *P*-values based on the test for a binomial proportion. Analyses were performed using SPSS version 20 (IBM Corp, Armonk, NY).

## Results

A total of 5994 (89% response rate) ED patients consented to participate in the study from southern Connecticut (Figure 1). The average time for survey completion was 6.2 minutes. The 3 EDs within the health care system are presented disaggregated and then were combined for purposes of analysis (Table 1). A total of 58.43% (3382/5788) of ED users were female; the mean age was 46 years old; whites comprised 42.14% (2410/5719), blacks 34.11% (1951/5719), and Latinos 23.75% (1358/5719) of the patient population; 2.95% (171/5788) of the participants elected to complete the survey in Spanish; 14.60% (845/5788) of the respondents had none to some schooling; and 39.38% (1775/4507) of the ED patients earned less than $15,000 per year. There was little if any variation among the 3 EDs, with the exception of income. A total of 47.10% (674/1431) of Saint Raphael’s patients earned less than $15,000 per year while only 34.97% (583/1667) of Yale-New Haven York Street Campus patients reported an income in this bracket (Table 1). ED patients reported high ownership of cell phones (4934/5788, 85.25%, *P*<.001) and mobile phones (2500/4934, 50.67%, *P*<.001) that were used for calling (4892/4934, 99.15%, *P*<.001). The older the patient, the less likely it was that the patient used their cell phone for texting (96% of 18-29 year olds vs 16% of those age 65 or older, *P*<.00). Ownership of iPhones (1093/2500, 43.72%) compared to Androids (1117/2500, 45.88%) were similar (*P*<.05). Of those patients with a contract, 49.57% (2446/4934) reported having unlimited minutes and 49.57% (2446/4934) reported having limited minutes. Furthermore, 20.25% (999/4934) of patients reported having a pay-as-you go plan, which may or may not have included a contract. Finally, 4.32% (213/4934) of patients reported owning a Medicaid phone (aka, “Obama phone”) (Table 2). Income was positively associated with mobile phone ownership (*P*<.001), use of health apps (*P*>.05), and use of personal health records (*P*<.001) (Table 3). Race played a significant role in texting and mobile phone ownership, with Hispanics reporting the highest rates (79% and 56%, respectively), followed by black non-Hispanics (77% and 54%, respectively) and white non-Hispanics (65% and 42%, respectively) (*P*<.05). ED users also demonstrated higher rates of African American and Latino patients (34% and 24%, respectively). In summary, ED patients had high rates of minorities, no to little education, and low income (Table 3).

While not directly comparable, as these 2 surveys are from 2 different sampling frames, the Pew Foundation and the California HealthCare Foundation (CHCF) conducted a similar media health care study during the same time period [99]. Coincidently, ED patients in our study had identical ownership of cell phones benchmarked against the Pew-CHCF study (4934/5788, 85.25%, *P*<.001). Income impacted the type of mobile phone and the nature of the contract, however, functionality remains identical. Basic functions such as calling (4892/4934, 99.15%, *P*>.05) and texting (3595/4935, 72.86%, CI 95% 72-74) were high (Table 2). Internet connections for browsing (2283/4934, 46.27%, 95% CI 45-48), e-mailing (2081/4934, 42.18%, 95% CI 41-44), and social networking (1903/4934, 38.57%, 95% CI 37-40) were less prevalent. Among all cell phone owners, 50.67% (2500/4934, 95% CI 49-52) reported that their device was a mobile phone. The Pew-CHCF study had a rate of 53%, meaning that ED users have 1% fewer mobile phones when benchmarked against the general population. iPhones were more pervasive among higher income ED patients than lower income patients; however, the functionality of Android phones is identical in terms of apps, texting, and Web browsing capability.

Table 3 demonstrates the health care utility for new media beyond calling capabilities according to selected demographic characteristics. The youngest age cohort of 18-29 years old reported the highest rates of texting (96%) compared to the older patients, significantly higher rates of mobile phones (79%, *P*<.001), high rates of using new media to seek health information (65%, *P*<.001). Among the eldest ED patients, those 65 years old or older, the highest rates were for using health apps (16%, *P*<.05) and seeking health information (33%, *P*<.001). African Americans (54%, *P*<.001) and Latinos (56%, *P*<.001) in the ED reported significantly higher rates of mobile phone ownership than whites (44%, *P*<.05). There was a similar pattern for seeking health information.

**Table 1:** Demographic breakdown of 3 emergency departments, July 12 to August 30, 2012.

	ED #1	ED #2	ED #3	Total
Demographic	Yale-New Haven Hospital York Street Campus	Yale-New Haven Hospital Saint Raphael Campus	Bridgeport Hospital	All EDs, combined (N=5788)
Female	1081/1922 (56.24%)	1177/1966 (59.87%)	1124/1900 (59.16%)	3382/5788 (58.43%)
Mean age, year (SD)	45 (18)	48 (21)	44 (19)	46 (20)
White, Non-Hispanic	891/1888 (47.19%)	889/1954 (45.50%)	630/1877 (33.56%)	2410/5719 (42.14%)
Black, Non-Hispanic	567/1888 (30.03%)	774/1954 (39.61%)	610/1877 (32.50%)	1951/5719 (34.11%)
Hispanic	430/1888 (22.78%)	291/1954 (14.89%)	637/1877 (33.94%)	1358/5719 (23.75%)
Spanish language survey	68/1922 (3.54%)	51/1966 (2.59%)	52/1900 (2.74%)	171/5788 (2.95%)
None to some schooling	253/1922 (13.16%)	304/1966 (15.46%)	288/1900 (15.16%)	845/5788 (14.60%)
Income <$15,000	583/1667 (34.97%)	674/1431 (47.10%)	518/1409 (36.76%)	1775/4507 (39.38%)

**Table 2:** New media use prevalence and taxonomies, July 12 to August 30, 2012.

		ED #1	ED #2	ED #3	Total
New media profile		Yale-New Haven Hospital York Street Campus	Yale-New Haven Hospital Saint Raphael Campus	Bridgeport Hospital	All EDs combined (N=5788)
Cell phone ownership		1677/1922 (87.25%)	1591/1966 (80.93%)	1666/1900 (88.68%)	4934/5788 (85.25%, 95% CI 84-86)
Cell phone use	Calling	1666/1677 (99.34%)	1572/1591 (98.81%)	1654/1666 (99.28%)	4892/4934 (99.15%, 95% CI 98.9-99.4)
	Texting	1235/1677 (73.64%)	1141/1592 (71.72%)	1219/1666 (73.17%)	3595/4935 (72.86%, 95% CI 72-74)
	E-mailing	654/1677 (39.00%)	624/1591 (39.22%)	803/1666 (48.20%)	2081/4934 (42.18%, 95% CI 41-44)
	Surfing Internet	762/1677 (45.44%)	652/1591 (40.98%)	869/1666 (52.16%)	2283/4934 (46.27%, 95% CI 45-48)
	Social networking	664/1677 (39.59%)	545/1591 (34.26%)	694/1666 (41.66%)	1903/4934 (38.57%, 95% CI 37-40)
	Playing games	422/1677 (25.16%)	430/1591 (27.03%)	564/1666 (33.85%)	1416/4934 (28.70%, 95% CI 27-30)
Mobile phone ownership		837/1677 (49.91%)	716/1591 (45.00%)	947/1666 (56.84%)	2500/4934 (50.67%, 95% CI 49-52)
Mobile phone operating system	iPhone	404/837 (48.27%)	278/716 (38.83%)	411/947 (43.40%)	1093/2500 (43.72%, 95% CI 42-46)
	Android	333/837 (39.78%)	333/716 (46.51%)	451/947 (47.62%)	1117/2500 (45.88%, 95% CI 43-47)
Mobile phone contract type	Contract, Limited Min	909/1677 (54.20%)	687/1591 (43.18%)	850/1666 (51.02%)	2446/4934 (49.57%, 95% CI 48-51)
	Contract, Unlimited Min	909/1677 (54.20%)	687/1591 (43.18%)	850/1666 (51.02%)	2446/4934 (49.57%, 95% CI 48-51)
	Medicaid phone (aka Obama phone)	66/1677 (3.94%)	84/1591 (5.28%)	63/1666 (3.78%)	213/4934 (4.32%, 95% CI 4-5)
	Pay-as-you-go	255/1677 (15.21%)	391/1591 (25.58%)	353/1666 (21.19%)	999/4934 (20.25%, 95% CI 19-21)

**Table 3:** New media device ownership and use by ED survey participants versus Pew-CHCF study data, July 12 to August 30, 2012.

Demographic (N=5788)		Text Messaging, 73% (CI 95% 72-74)			Mobile Phone Ownership, 51% (CI 95% 49-52)			Use of Health Apps, 19% (CI 95% 17-21)			Health Info Seeking, 60% (CI 95% 58-62)			Personal Health Records, 6% (CI 95% 6-8)
		EDs	Pew-CHCF	*P*	EDs	Pew-CHCF	*P*	EDs	Pew-CHCF	*P*	EDs	Pew-CHCF	*P*	EDs
Gender	Men	68 (66-71)	81	<.001	47 (45-49)	46	>.05	17 (15-20)	16	>.05	53 (49-56)	29	<.001	7 (6-8)
	Women	76 (74-77)	80	<.001	53 (52-55)	45	<.001	20 (18-22)	23	.007	64 (62-67)	33	<.001	6 (5-7)
Age	18-29	96 (95-97)	97	.04	79 (77-81)	66	<.001	18 (16-21)	24	<.001	65 (62-68)	42	<.001	7 (5-8)
	30-49	84 (82-85)	92	<.001	54 (52-57)	59	<.001	20 (18-23)	19	>.05	57 (53-60)	39	<.001	8 (7-9)
	50-64	55 (52-58)	72	<.001	30 (27-33)	34	.004	17 (11-22)	16	>.05	53 (47-60)	19	<.001	7 (5-8)
	65+	16 (13-19)	34	<.001	10 (7-12)	11	>.05	16 (4-27)	10	>.05	33 (19-47)	9	<.001	2 (1-2)
Race/Ethnicity	White, Non-Hispanic	65 (63-67)	79	<.001	44 (42-46)	42	>.05	21 (18-24)	19	>.05	61 (58-65)	27	<.001	8 (6-9)
	Black, Non-Hispanic	77 (75-79)	80	.008	54 (51-56)	47	<.001	17 (15-20)	21	.01	57 (54-61)	35	<.001	5 (4-5)
	Hispanic	79 (77-82)	85	<.001	56 (53-59)	49	<.001	18 (15-21)	15	.03	61 (57-65)	38	<.001	6 (5-7)
Annual Household Income	<$30,000	71 (69-73)	78	<.001	45 (43-47)	35	<.001	16 (14-18)	14	>.05	63 (60-66)	28	<.001	3 (3-4)
	$30,000-$59,999	76 (73-79)	78	>.05	56 (53-60)	42	<.001	21 (17-25)	21	>.05	59 (54-64)	30	<.001	8 (6-10)
	$60,000-$89,999	78 (74-82)	89	<.001	60 (56-65)	56	>.05	23 (17-28)	21	>.05	61 (54-67)	37	<.001	11 (8-14)
	≥$90,000	80 (76-85)	90	<.001	70 (65-74)	68	>.05	26 (20-32)	23	>.05	67 (60-73)	37	<.001	22 (18-26)
Education Level	No HS Diploma	55 (51-59)	65	<.001	33 (29-37)	21	<.001	------	Not reported		54 (47-62)	17	<.001	2 (1-3)
	High School Graduate	69 (67-71)	75	<.001	42 (39-44)	36	<.001	14 (11-16)	11	.04	55 (51-58)	26	<.001	3 (3-4)
	Some College	83 (80-85)	85	.01	62 (60-65)	50	<.001	20 (17-24)	24	.02	62 (59-66)	33	<.001	9 (7-11)
	College+	78 (76-81)	86	<.001	63 (61-66)	61	>.05	24 (21-28)	22	>.05	65 (61-69)	38	<.001	15 (13-18)

## Discussion

### Principal Findings

While the conventional main focus of hospital EDs has been to provide immediate treatment to patients with acute conditions, the use of new media could extend the reach of the ED visit. These clinical encounters provide unique and important opportunities to the clinicians and system of health care to positively influence individual health behavior beyond the emergency department setting.

We sought to define and differentiate new media from cell phone ownership to bring health care operationalization of electronic devices consistent with the communication literature. Furthermore, because information technology is already playing an increasing role in improving health care, delivering interventions, navigating the health care system, and improving the public’s health at large, we wanted to determine (beyond the anecdotal) that sufficient numbers of ED patients own and use new media. Survey participants’ ownership of cell phones (4934/5788, 85.25%) and device usage for calling (4892/4934, 99.15%) and texting (3595/4935, 72.86%) were high. Among all cell phone owners, mobile phone ownership was moderate (2500/4934, 50.67%) with minorities reporting the highest rate of ownership. Benchmarked against the Pew-CHCF study [99], we observed similar prevalence figures for cell phone ownership and use for texting as well as mobile phone ownership (Table 3).

EDs are concerned with enhancing continuity of care throughout an entire health system and optimizing cost containment. As a result, they have generally heightened and expanded their attention to pre-hospital and post-discharge care implications of acute care. Finding new forms of effective communication facilitates expanding the scope of prevention, health promotion, health maintenance, and disease management services [100-102].

ED patients are segmented in this study to those most likely to own and use new media technology. Hence, we determined the characteristics of ED patients that own and use new media to tailor intervention strategies. Text messaging can be used to provide health information to most cell phone users (depending on their phone plan). We examined the relationship between ED patients’ use of text messaging and individual patient characteristics. A higher prevalence of text messaging was reported by ED patients who were female, younger, nonwhite, and more educated (Table 2). Text messaging was less common among ED patients regardless of gender, age group, race/ethnicity, or socioeconomic status.

While mobile phone ownership is not as ubiquitous as overall cell phone ownership, mobile phone technology is important for behavioral interventions (eg, mobile phone health apps). Thus, we examined the relationship between ED patients’ mobile phone ownership and individual patient characteristics. Participants who were female, younger, nonwhite, and had higher income reported greater ownership of mobile phone technology (Table 3). Notably, ED participants with lower household income, less formal education, and either urban or rural residency (data not shown) reported the highest ownership.

Female and younger ED patients who owned mobile phones, as well as those with greater educational attainment, reported higher online searching for health or medical information. Consistently, ED participants reported greater health information seeking than the general population as measured by Pew-CHCF.

We found similar patterns of usage of cell and mobile phones in both the ED patient population and the general population with the exception that ED patients are more likely to use desktop and laptop computers to seek health information than a mobile phone and the general population is more likely to rely on e-mail to communicate through a laptop or desktop computer.

### Limitations

We compared the prevalence of health information seeking by ED patients with that of the general population. ED participants invariably reported greater health information seeking than participants in the Pew-CHCF survey. Individuals presenting to the emergency department likely have health conditions that trigger new media use to manage disease and seek information on treatment and care.

Racial/ethnic minorities and persons of lower socioeconomic status were overrepresented in the EDs as compared to the general US catchment area. Compared to the benchmark Pew-CHCF survey, our ED sample was similar in terms of gender but (predictably) was made up of more nonwhite participants who were poorer and had less schooling.

### Conclusions

Our study formally defines new media and disambiguates cell phones from mobile phones. We established a scientifically derived baseline of new media use for ED patients and determined that a critical mass of patients use new media and would perhaps benefit from new media technology to manage their health and seek information. Most importantly, we found that more marginalized populations—such as the poor, homeless [48], and minority patients—do not differ significantly in ownership or usage rates from the general population and that sufficient ownership exists to reach a significant portion of the population using new media. New media may be a health care equalizer to address health care disparities by reaching minorities and low income patients better. This research also suggests that potentially assisting ED patients without information technology is an option to extend services such as the Lifeline Program for Low-Income Consumers [103]. This study increases confidence in the utility of new media for health care services, interventions, and follow up [61,104 105].

## Abbreviations

ED: emergency department
CHCF: California HealthCare Foundation
CI: confidence interval
RA: research assistant
YNHHS: Yale-New Haven Health System
